# Movement-Based Priming: A Clinical Trial on the Effect of Cross-Training on Locomotor Abilities of Children with Unilateral Cerebral Palsy

**DOI:** 10.3390/children12040508

**Published:** 2025-04-15

**Authors:** Tamer M. Elsaeed, Ragab K. Elnaggar, Mohammed F. Elbanna, Mshari Alghadier, Aziza M. Kamel, Ahmed M. Aboeleneen, Fahad A. Qissi, Marwa M. Ismaeel

**Affiliations:** 1 Department of Physical Therapy for Pediatrics, Faculty of Physical Therapy, Cairo University, Giza 12611, Egypt; 2 Department of Physical Therapy, Faculty of Medical Rehabilitation Sciences, King Abdulaziz University, Jeddah 22252, Saudi Arabia; 3 Department of Health and Rehabilitation Sciences, College of Applied Medical Sciences, Prince Sattam Bin Abdulaziz University, Abdullah Ibn Amer St., Al-Kharj 11942, Saudi Arabia; 4 Faculty of Physical Therapy, Cairo University, Giza 12611, Egypt; 5 Medical Surgical Nursing, College of Nursing Sciences, Prince Sattam Bin Abdulaziz University, Al-Kharj 11942, Saudi Arabia; 6 Physical Therapy and Health Rehabilitation, Comprehensive Rehabilitation Center, Al-Kharj 16432, Saudi Arabia

**Keywords:** cerebral palsy, movement-based priming, neural plasticity, strength, mobility, locomotion

## Abstract

**Background:** Children with unilateral cerebral palsy (UCP) frequently experience limitations in locomotor abilities, attributable to a complex interplay of factors including muscle weakness and reduced joint mobility. Movement-based priming, such as cross-training (CT), has emerged as a potential intervention to enhance motor function in children with UCP. However, evidence of its efficacy remains limited. Objective: This study aimed to investigate the effect of CT—specifically, a strengthening-based unilateral priming protocol—on muscle strength, joint mobility, and locomotor abilities in children with UCP. **Methods:** Thirty-six children with UCP were enrolled in a randomized controlled trial. Participants were randomized into two groups: the control group (*n* = 18; underwent a tailored physical rehabilitation program) and the CT group (*n* = 18; participated in a CT program incorporating unilateral priming exercises targeting the less-affected lower followed by the same rehabilitation program administered to the control group). Dorsiflexor strength, ankle joint mobility, and locomotor ability assessed via the 10 m walk test (10 mWT), 6 min walk test (6 MWT), and timed up-and-go test (TUG) were evaluated pre- and post-intervention. **Results:** Significant moderate-to-large between-group differences were observed in dorsiflexor strength (*p* = 0.032, partial η^2^ = 0.128), ankle mobility (*p* = 0.016, partial η^2^ = 0.159), and locomotor ability (10 mWT [*p* = 0.017, partial η^2^ = 0.157]; 6 MWT [*p* = 0.004, partial η^2^ = 0.222]; TUG [*p* = 0.047, partial η^2^ = 0.111]). The CT group demonstrated superior improvements concerning all outcomes. **Conclusions:** Unilateral priming through strengthening-based CT is a viable intervention for enhancing motor function in children with UCP, providing a promising complement to the current rehabilitation protocols.

## 1. Introduction

Cerebral palsy (CP) represents a group of neurological conditions that disrupt the typical development of motor function and posture, stemming from brain injury or malformations occurring early in the developmental process. This injury or malformation can occur before, during, or shortly after birth, or in the early years of life. Key risk factors include premature birth, low birth weight, multiple births, and maternal infections during pregnancy [1,2]. Additionally, complications during birth, such as placental detachment or problems with the umbilical cord, can disrupt the oxygen supply to the baby, leading to CP [1]. In some cases, CP can also be acquired due to brain damage from infections like meningitis or encephalitis, or from head injuries [2]. It is a leading cause of childhood motor disability, with a global prevalence ranging from 1.5 to over 4 per 1000 live births [3,4]. Importantly, CP often coexists with other neurological comorbidities, with epilepsy being one of the most prevalent. Research indicates that epilepsy—a condition often associated with underlying structural brain abnormalities, such as periventricular lesions or cortical malformations—occurs disproportionately across CP subtypes, with prevalence rates varying by motor severity and brain lesion type, reaching up to 49% in some cohorts [5]. Epilepsy can further exacerbate motor and cognitive dysfunction, either through the direct effect of seizures or as a consequence of anti-epileptic drug side effects. These factors pose additional challenges for therapeutic interventions aimed at enhancing motor function in this population. The spectrum of CP encompasses a variety of subtypes, each characterized by distinct patterns of movement limitations [6]. Unilateral CP (UCP) stands as a prominent subtype that primarily affects one side of the body, resulting in unilateral motor impairments. Epidemiological studies consistently demonstrate a substantial prevalence of UCP, constituting approximately 33–39% of the overall CP population [7].

Children with UCP face a multifaceted array of motor challenges, including impaired strength, reduced flexibility, and difficulties with locomotion [8]. Muscle weakness, often more pronounced on the affected side, can significantly impact the ability to generate force for activities such as lifting, reaching, and walking [9,10]. Moreover, limitations in joint range of motion, frequently caused by muscle contracture and spasticity, further restrict movement and can hinder functional abilities [11]. These motor impairments, coupled with difficulties with balance and coordination, can significantly impact the child’s ability to walk, run, and participate in age-appropriate activities, thus impacting the overall quality of life [12,13]. Therefore, continuous research is crucial to discover the most effective rehabilitation approaches for addressing these impairments. This research should focus on leveraging the potential of these approaches to stimulate neuroplasticity, enhance motor learning, and facilitate functional gain in children with UCP.

Clinical practice for children with UCP involves multifaceted exercise approaches to improve muscle strength, joint mobility, and locomotor abilities. This typically encompasses progressive resistance, plyometric, balance, Pilates, and functional exercises [14,15,16,17,18,19,20]. However, current practices often exhibit a unilateral focus, predominantly targeting the affected limbs while potentially neglecting the contribution of the less affected limbs. In children with UCP exhibiting significant lower-limb weakness, the implementation of direct strength-training exercises can present significant challenges. These challenges arise from the inherent difficulties in effectively engaging and strengthening severely weakened muscles, potentially leading to limited functional gains, increased psychological burden, and feelings of inadequacy [21]. In this context, the exploration of alternative approaches that maximize engagement while also minimizing the potential barriers remains necessary.

The concept of movement-based priming posits that the utilization of continuous bodily motion can serve to augment the efficacy of subsequent primary interventions [22]. Priming movements can exhibit a range of characteristics, including laterality (bilateral or unilateral), activity level (active or passive), and joint involvement (single or multi-joint). The application of priming movements can encompass various exercise modalities, such as aerobics, strengthening, and balance exercises [23,24]. Movement-based priming, unlike motor training, which emphasizes the acquisition of specific motor skills, typically involves repetitive movements that are not explicitly designed to achieve a particular motor skill [22]. Cross-training (CT), a form of unilateral priming, has gained considerable attention within the field of physical rehabilitation [25,26]. CT entails promoting muscle activity on the affected side through the application of resistance to the homologous muscles on the less-affected side [27]. Although the underlying mechanism of CT remains to be fully elucidated, it may involve a distributed network within the frontal cortex responsible for motor planning and execution. This network, with its interhemispheric connections, suggests that motor learning in one hemisphere could potentially influence motor activity in the contralateral hemisphere, contributing to the observed effects of CT [28].

Prior research has explored the effects of CT in the adult population with hemiplegia, with promising results in enhancing muscle strength, improving motor function, and facilitating functional independence [25,29,30,31]. However, the potential benefits of such a training approach in children with UCP remain largely unexplored. Given the unique neurodevelopmental trajectory and motor challenges faced by this population, investigating the efficacy of CT in this context is crucial. Thus, the present study was designed to explore the efficacy of CT on muscle strength, joint mobility, and locomotor abilities in children with UCP.

## 2. Materials and Methods

### 2.1. Design Overview

This study, designed as a two-arm randomized controlled trial, carried out between July 2023 and January 2024, followed ethical principles outlined in the Declaration of Helsinki and was approved by the local ethics committee at the Faculty of Physical Therapy, Cairo University, Egypt (approval number: P.T.REC/012/004588, dated 11 June 2023). Written informed consent was obtained from parents/legal representatives of all children prior to their inclusion in the study. The trial protocol has been registered with ClinicalTrials.gov under the identifier NCT06750081. In this study, a single-blind protocol was adopted, ensuring the assessor remained blinded to treatment allocation, was not engaged in treatment implementation, and played no role in data analysis.

### 2.2. Participants

A total of 36 children with a confirmed diagnosis of spastic UCP [32], aged between six and eight years (targeting a developmental stage where motor learning and neural plasticity are highly active) [33], were recruited from the various local referral hospitals/rehabilitation centers to participate in this study. Participants exhibited spasticity levels ranging from grade 1 to 1+ as assessed by the Modified Ashworth Scale [34]. The inclusion criteria required that all children were capable of standing and walking autonomously without the use of assistive devices. However, children who routinely used ankle–foot orthoses were eligible for participation, provided they could ambulate without additional aids. Additionally, participants were required to demonstrate the ability to comprehend and follow verbal instructions during both testing and training sessions, ensuring compliance with the study protocol and accurate execution of tasks. Children were excluded if they presented with fixed musculoskeletal deformities, significant cognitive or perceptual impairments, or uncorrected visual or auditory deficits. Additional exclusion criteria included comorbid conditions that could affect safety or compliance during assessment or interventions, such as epilepsy or severe behavioral disorders. Furthermore, participants were ineligible if they were currently using muscle relaxants or other neurolytic agents, or had received botulinum toxin injections within the previous six months.

#### 2.2.1. Sample Size

A priori power analysis was conducted using G*Power software (version 3.1.9.4 for Windows). The analysis was performed for a two-group comparison, with an assumed effect size (Cohen’s d) of 0.8, a two-tailed significance level (α) of 0.05, and desired statistical power of 80%. The calculation revealed that a minimum of 15 participants per group (total *n* = 30) would be required to achieve the target power. To account for the potential attrition and ensure the study’s reliability, 18 participants per group (total *n* = 36) were enrolled, which resulted in an achieved power of 80.5%.

#### 2.2.2. Randomization and Intervention Assignment

To ensure equitable distribution of participants across the study groups, a stratified randomization approach was adopted. Following the initial assessment, participants were divided into strata based on gender and spasticity level. Within each stratum, they were randomly assigned to either the control group or the CT group using a computer-generated randomization sequence. Children were divided into two groups, each comprising 18 participants. The control group underwent a tailored physical rehabilitation program. The CT group participated in a CT program that incorporated movement-based priming exercises targeting the less-affected lower limb, followed by the same physical rehabilitation program administered to the control group, with a 5–10 min rest period between sessions. The intervention included approximately 30 h of exercise training, delivered in one-hour sessions five times per week over six consecutive weeks. To uphold the rigor and consistency of the intervention protocol, participants who failed to attend more than two successive sessions were disqualified from the study.

### 2.3. Outcome Measures

This study featured an integrated assessment protocol to characterize impairment and functional parameters in children with UCP. The evaluation matrix comprised quantitative metrics of muscle strength, joint mobility, and locomotor abilities. Assessments were conducted by the same researcher at two time points: pre- and post-intervention.

#### 2.3.1. Muscle Strength

Dorsiflexor strength on the affected side was quantified using a handheld dynamometer (HHD; Lafayette Instrument, Lafayette, IN, USA). Participants were seated with the affected leg supported on a plinth, ensuring knee extension and neutral ankle position. The HHD was positioned on the dorsal surface of the foot near the metatarsal heads. Participants were instructed to perform maximal isometric contraction against the HHD, with resistance applied perpendicular to the foot. Peak force, recorded in newtons (N), was measured across three trials, separated by 30 s rest periods, and the mean value was calculated for the analysis. Rigorous adherence to consistent positioning, instruction delivery, and standardized HHD calibration procedures was ensured. This methodological approach is based on previous research demonstrating the reliability of HHD for muscle strength assessment [35].

#### 2.3.2. Joint Mobility

The active range of motion (AROM) of ankle dorsiflexion was evaluated using a universal goniometer, with participants positioned supine on a plinth. The pelvis and trunk were stabilized to prevent compensatory motion. The goniometer axis was aligned with the lateral malleolus, with the stationary arm positioned along the fibula’s lateral midline and the moving arm aligned with the fifth metatarsal’s lateral midline. Participants were instructed to actively dorsiflex the ankle to their maximum range, with the examiner ensuring stability of the knee and leg throughout the assessment. The maximum AROM achieved was recorded in degrees. Three trials were performed, and the mean value was computed and utilized for data analysis. This approach complies with established guidelines and is supported by published research highlighting the validity and reliability of these measures for children with CP [36].

#### 2.3.3. Locomotor Ability

Three key assessments were administered to measure different dimensions of locomotor abilities: the 10-m walk test (10 mWT), focusing on preferred walking speed [37]; the 6-minute walk test (6 MWT), targeting endurance and functional capacity [38]; and the modified timed up-and-go test (TUG), evaluating functional mobility and balance [39].

For the 10 mWT, a 14 m walkway was prepared, incorporating 2 m acceleration and deceleration zones at both ends of the central 10 m measured distance. Participants were instructed to walk the 10 m section at a self-selected, comfortable pace. Timing commenced when the first foot crossed the start line of the measured section and concluded when either foot crossed the end line. A digital stopwatch was used to record the time, and walking speed was calculated in meters per second (m/s). Three trials were conducted, with the average value utilized for analysis. This test is a well-established and reliable method for assessing ambulation performance as corroborated by the literature [37].

The 6 MWT was administered following standardized procedures to assess endurance and functional capacity. A clearly marked 30 m rectangular walkway with a hard unobstructed surface was used. Participants were instructed to walk as far as possible within six minutes, with the option to stop and rest if needed, though they were encouraged to continue walking once able. They received standardized verbal instructions and motivational statements at one-minute intervals throughout the test. The total distance walked was measured using a distance-measuring wheel. The use of walking aids, orthotics, or any adaptive strategies during the test was documented [38].

In the modified TUG test, participants were seated on a stable stool with their feet flat on the floor and instructed to reach for a star located three meters away on the wall. They were allowed to put on footwear or orthotics; however, no additional physical supports were provided. Unlike the conventional TUG test, participants were permitted to pause briefly for rest (without sitting), and it was emphasized that this task was not a race. The timing began as participants stood up from the stool and concluded as they returned to the seated position, capturing only “movement time”. The examiner provided clear instructions, including details on the timing procedure. After one practice trial, three trials were performed, and the average time was recorded [39].

### 2.4. Interventions

#### 2.4.1. Physical Rehabilitation Program

This study implemented a structured physical rehabilitation regimen, uniformly administered to the control and CT group participants, with an emphasis on enhancing mobility and functional outcomes. The program prioritized four objectives: (a) addressing atypical motor patterns, (b) restoring/maintaining flexibility, (c) augmenting proprioceptive awareness, and (d) refining gait biomechanics. The program was personalized to accommodate the distinct needs and limitations of each participant while maintaining a standardized structure to ensure uniformity and facilitate cross-participant comparisons. The program was conducted in one-hour sessions, five times a week for six consecutive weeks. Key components included the following:

- *Flexibility exercises*: A daily regimen of stretching exercises was implemented focusing on muscle groups prone to tightness, including plantar flexors, hamstrings, and hip adductors. Each stretch was maintained for 30 s, performed in three sets, with 30 s intervals for muscle release.
- *Strengthening exercises*: Targeted exercises aimed at fortifying the muscles around the hip, knee, and ankle were administered. These exercises were performed in functional contexts, employing both open and closed kinetic chain methods. Resistance was tailored to individual needs, either manually or with resistance bands.
- *Weight*-bearing activities: These exercises aimed to bolster weight acceptance and postural stability, while ensuring that participants maintained comfortable and supported stances. Exercises focused on enhancing weight-bearing capabilities of the affected limb, incorporating tasks like sit-to-stand, standing weight-shifting (side-to-side; front-to-back), single-leg stance, tandem stance, standing step-ups, and standing marches.
- *Gait training*: Utilizing an open-environment setting, gait training focused on walking at various speeds and overcoming different challenges. Key aspects included enhancing gait dynamics, with verbal cueing and feedback being crucial elements of the training.

#### 2.4.2. Cross-Training

Participants in the CT group also took part in an exercise-based priming CT program targeting the less-affected side to promote neural plasticity [22,28]. The intervention, delivered by a pediatric physical therapist, was scheduled 10 to 15 min before the standardized physical rehabilitation program commenced. Specifics of the program are as follows:

- *Exercises*: The CT protocol incorporated resisted exercises targeting four specific muscle groups of the less-affected limb: knee flexors, knee extensors, ankle dorsiflexors, and plantar flexors. The exercise comprised isometric, concentric, and eccentric contraction resistance, each for two seconds.
- *Exercise tailoring*: A priori assessment was conducted to determine the optimal training repetitions/sets that each child was able to perform and resistance forces. Exercises were performed at an intensity corresponding to 4–6 on the Borg CR-10 scale for perceived exertion rating [40], such that each participant was able to perform 10 repetitions. This approach allowed the optimization of therapeutic benefits and participant comfort while also minimizing the risk of overexertion or injury.
- *Sets and repetitions*: The protocol allowed for a maximum of 30 repetitions per muscle group, structured as three sets of 10 repetitions, with standardized rest intervals of 2–3 min between sets.

To optimize performance, participants engaged in a 5 min warm-up, before the primary exercise set, beginning with three minutes of aerobics (e.g., slow waking or unloaded cycling) followed by two minutes of dynamic movements such as leg swings, marching in place, and partial squats. To facilitate recovery, a 5 min cool-down was performed post-exercise, starting with three minutes of light aerobic activities and ending up with two minutes of static stretching targeting the quadriceps, hamstrings, and calves.

### 2.5. Statistical Analysis

All statistical analyses were conducted using SPSS, version 26 (IBM Corp., Armonk, NY, USA). The Shapiro–Wilk test was employed to assess the normality of data distribution, ensuring the suitability of parametric tests for the subsequent analyses. Multivariate analysis of variance (MANOVA) was utilized to examine differences between groups across dependent variables as a unified set, with assumptions of homogeneity of variance–covariance matrices and the absence of multicollinearity verified. Then, a follow-up univariate analysis (ANOVA) was performed to identify which dependent variables exhibited significant group differences. When significant between-group differences were detected, within-group changes were further evaluated using paired t-tests. Effect sizes were quantified to provide a comprehensive understanding of the magnitude of observed effects: partial η^2^ was reported for ANOVA models, with values interpreted as small (η^2^ ≥ 0.01), medium (η^2^ ≥ 0.06), or large (η^2^ ≥ 0.14), while Hedges’ g was computed to estimate the effect sizes for within-group changes, interpreted as small (g ≥ 0.20), medium (g ≥ 0.50), or large (g ≥ 0.80). A significance level of *p* < 0.05 was consistently applied across all tests.

## 3. Results

### 3.1. Participant Enrollment and Retention

**Figure 1:**
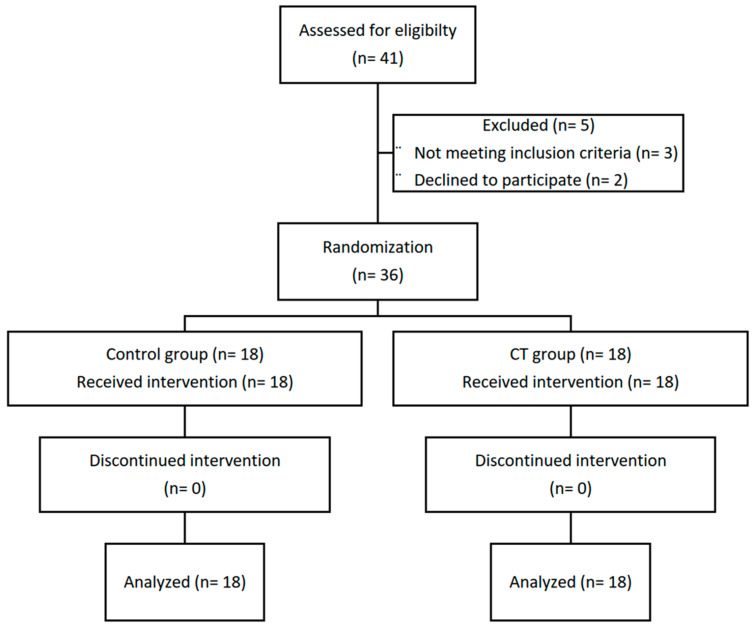
A flow diagram illustrating the progression of the study participants throughout the study.

Figure 1 details the progression of participants through the study. Forty-one children with UCP were initially screened for eligibility. Five children were excluded: three due to not fulfilling the inclusion criteria and two due to declining participation. The remaining 36 participants were randomly allocated to the control group or the CT group (*n* =18 per group). All participants completed their assigned interventions. No participants withdrew or were excluded due to non-compliance (operationalized as missing more than two consecutive or separate sessions) or failure to complete post-intervention assessments.

### 3.2. Baseline Homogeneity

Table 1 presents a summary of the baseline demographic and clinical characteristics, along with the pre-intervention measures of all pendent outcomes. The control and CT groups demonstrated homogeneity with respect to age, sex distribution, anthropometry, affected limb, and spasticity (*p* > 0.05). Furthermore, the two groups exhibited no significant differences in pre-intervention measures of the dependent variables (i.e., dorsiflexor strength, AROM, and locomotor ability [10 mWT, 6 MWT, and TUG]) (*p* > 0.05).

### 3.3. Differential Effects of Intervention

The MANOVA revealed a significant overall multivariate effect of group (Wilks’ λ = 0.257, F_10,25_ = 7.230, *p* < 0.001, partial η^2^ = 0.743), indicating that the intervention had a substantial effect on the dependent variables (i.e., strength, AROM, 10 mWT, 6 MWT, and TUG), considered as a set. More specifically, the multivariate analysis indicates that ~74% of the variance in the dependent variables is associated with the interventions.

The follow-up univariate ANOVAs revealed significant medium-to-large differences between the CT and control group in terms of dorsiflexor strength (F_1,34_ = 4.986, *p* = 0.032, partial η^2^ = 0.128) and AROM (F_1,34_ = 6.433, *p* = 0.016, partial η^2^ = 0.159). The CT group demonstrated greater improvement in both outcomes compared to the control group, suggesting the potential therapeutic advantages of CT (Table 2). Furthermore, significant medium-to-large group differences were observed for locomotor ability variables, including the 10 mWT (F_1,34_ = 6.323, *p* = 0.017, partial η^2^ = 0.157), 6 MWT (F_1,34_ = 9.727, *p* = 0.004, partial η^2^ = 0.222), and TUG (F_1,34_ = 4.256, *p* = 0.047, partial η^2^ = 0.111). The CT group consistently exhibited superior performance across the three measures compared to the control group (Table 3).

## 4. Discussion

This study sought to explore the efficacy of CT as a priming strategy in addressing muscle strength, joint mobility, and locomotor function deficits in children with UCP. The results, in support of the initial hypothesis, revealed that CT had a significant positive impact on these rehabilitation outcomes. Specifically, participants in the CT group exhibited enhanced strength of ankle dorsiflexors and increased AROM of the ankle joint, particularly on the affected limb. The locomotor performance also showed marked progress in the CT group participants, with faster gait speeds in the 10 mWT, longer distance coverage in 6 MWT, and improved balance and functional mobility in the TUG test. These results, therefore, highlight the potential of CT as a movement-based priming strategy to enhance motor readiness and support rehabilitation outcomes in children with UCP.

However, it is worth mentioning that, since the study participants had mild spasticity (ranging from grade 1 to 1+ on the Modified Ashworth Scale), the observed improvement through the applied rehabilitation programs, including CT, might have been enhanced. At these spasticity levels, there is only a slight increase in muscle tone, which allows muscles to be more responsive to exercises. This increased responsiveness likely contributed to better outcomes in motor function and mobility, as muscles are less resistant to movement and more adaptable to therapeutic interventions [10].

While the present study does not delve into the mechanisms that account for the observed improvements, a credible explanation may be put forth. The CT priming strategy focusing on the less-affected limb likely leverages interlimb transfer and neural plasticity mechanisms to drive improvements in strength, mobility, and locomotor performance [22,41]. This phenomenon, often referred to as cross-education or contralateral strength transfer, is mediated by the bilateral organization of the motor cortex, where training one limb induces adaptation in the homologous muscles of the opposite limb through a shared neural pathway [27,41,42]. Specifically, engaging the less-affected limb in repetitive activities may enhance excitability in the ipsilateral motor cortex, which also controls the affected limb, thereby promoting cortical reorganization and improving motor output to the impaired side [43,44]. Additionally, CT may activate subcortical structures such as the cerebellum and basal ganglia, which play a critical role in coordinating motor commands across both sides of the body [43], further facilitating bilateral motor gain. From a biomechanical perspective, strengthening the less-affected side can improve compensatory strategies during ambulation, reducing the over-reliance on the affected limb while simultaneously encouraging its gradual engagement through a load-sharing mechanism [27]. Furthermore, proprioceptive feedback generated during CT of the less-affected limb may enhance sensory–motor integration, contributing to improved balance, postural control, gait symmetry, and overall locomotor abilities [10,27,41]. The psychological benefits of focusing on the less-affected limb should not be overlooked, as it may boost confidence and motivation by providing a sense of mastery and control, which can translate to greater effort and participation during rehabilitation tasks involving the affected side [21]. Together, these mechanisms underscore the potential of targeting the less-affected limb as a strategic entry point for priming interventions, introducing a novel effective approach to indirectly enhance motor function in the affected limb and promote holistic improvements in locomotor abilities for children with UCP.

As the literature currently stands, there is a conspicuous lack of investigation into the use of CT as a priming strategy in children with UCP, making direct comparability of the data demonstrated herein somewhat challenging. This oversight is notable considering that early intervention strategies are crucial in optimizing developmental outcomes in the pediatric population [45]. While numerous studies have focused on motor learning, task-specific, and functional training [14,15,16,19,46,47], the synergetic effects of CT—known to enhance motor performance and functional recovery in other populations [47]—have remained unexplored in this context. The present study addresses this important gap, providing empirical evidence that CT can serve as a valuable component of rehabilitation (i.e., as a preparatory strategy to optimize task-specific rehabilitation), thereby enriching the therapeutic landscape for children with UCP.

Despite the lack of direct evidence in children with UCP, studies involving adult stroke patients provide valuable insights. Stroke survivors, who share similarities in clinical presentation with children with UCP—such as hemiparesis and disrupted motor control—have demonstrated improved outcomes when CT is used as a preparatory strategy for task-specific rehabilitation activities. These benefits have been observed across multiple domains, including reduction in motor impairments, enhancements in balance and coordination, and improvements in functional performance [29,47,48]. Notably, the mechanisms underlying these effects, such as neural plasticity, bilateral activation of motor networks, and interhemispheric communication, are also relevant to the pediatric population [49]. The results of the present study align with these earlier findings, demonstrating that CT can effectively prime the nervous system in children with UCP, leading to measurable improvements in motor skills and functional performance. Our data corroborate the transferability of the CT principles from adult stroke recovery to pediatric rehabilitation, reinforcing the potential of this approach to complement existing therapies and optimize rehabilitation outcomes in children with UCP.

### 4.1. Strengths and Limitations

This study holds several important strengths that bolster the validity and clinical significance of its results. Notably, it is the first to investigate the impact of CT as a priming strategy in children with CP, addressing a critical gap in the literature and providing insights into innovative therapeutic approaches. The adoption of a randomized controlled single-blind methodology further enhances the credibility of the results by reducing bias and ensuring rigorous comparison between the intervention (i.e., CT) and control groups. Moreover, the study’s emphasis on locomotor abilities, besides strength and mobility, reflects the priorities of children with CP and their families, focusing on functional mobility—a domain that directly influences daily life and overall well-being. That is, prioritizing outcomes that resonate with real-world challenges enhances the ecological validity of the study’s results and underscores the translational value of CT as a meaningful intervention. These methodological and conceptual strengths establish a solid foundation for future research that seeks to optimize rehabilitation strategies for children with UCP.

The results of this study, however, should be interpreted in light of several limitations that may influence the generalizability. First, the lack of comparable data from children with CP or those with other neuromotor impairments restricts the ability to contextualize the outcome within a broader population, potentially limiting the applicability of the results to other clinical groups. Second, the relatively small sample size may reduce the statistical power and limit the robustness of the conclusions, particularly in detecting subtle effects or subgroup differences. Additionally, the age range of participants was confined to 6–8 years, which may not capture the developmental variations or treatment responses in younger or older children. Finally, the absence of long-term follow-up after the treatment period hinders the ability to assess the durability of the observed effects and their potential impact on developmental trajectories. Future research should address these limitations by including larger, more diverse cohorts, extending the age range, and incorporating longitudinal assessments to evaluate the sustained efficacy of the intervention.

### 4.2. Clinical and Research Implications

The findings of the current study carry significant clinical and research implications. The significant improvements in strength, mobility, and locomotor abilities observed in this study highlight the potential of CT as a movement-based priming intervention to enhance outcomes of standard rehabilitation care for children with UCP. Utilizing CT as a percussor to standard affected-limb-oriented exercises could prime the motor system, enhancing readiness and amplifying the effectiveness of subsequent rehabilitation activities, thus offering a cutting-edge strategy to maximize functional gains in this population. Beyond physical improvements, the priming effect of CT could also facilitate broader developmental benefits such as increased participation in social and recreational activities, emphasizing the importance of integrating holistic intervention frameworks that address both motor and psychosocial well-being. These results provide compelling evidence for the role of CT in promoting neural plasticity and motor learning and suggest that diverse preparatory activities can prime the neuromotor system for more effective engagement in rehabilitation tasks. Future research could explore optimal protocols for combining CT with standard care, including dosing, timing, and activity selection, while also investigating its applicability in other pediatric populations with motor impairments. Additionally, examining secondary outcomes such as quality of life and psychosocial development could further elucidate the comprehensive impact of this priming strategy, paving the way for more effective, integrative rehabilitation paradigms for children with UCP.

## 5. Conclusions

The results of this study underscore the transformative potential of CT as a movement-based priming strategy for children with UCP, with significant gains in strength, mobility, and locomotor performance. These results support the incorporation of CT into the rehabilitation program to enhance motor readiness and foster functional outcomes. Future research should focus on refining protocols, exploring long-term effects, and expanding application to other populations, ultimately clearing the path for more effective, personalized rehabilitation strategies that empower children to achieve their full motor potential.

## Figures and Tables

**Table 1:** Baseline assessment of demographic, clinical, and anthropometric variables, and dependent outcomes across study groups.

	Control Group (*n* = 18)	CT Group (*n* = 18)	*p*- Value
Demographic, clinical, and anthropometric traits			
Age, years	7.02 ± 0.44	7.07 ± 0.61	0.76 ^‡^
Gender (b/g), *n* (%)	11 (61.1)/7 (38.9)	10 (55.6)/8 (44.4)	0.11 ^§^
Side affected (RT/LT), *n* (%)	8 (44.4)/10 (55.6)	6 (33.3)/12 (66.7)	0.47 ^§^
MAS level (1/1+), *n* (%)	10 (55.6)/8 (44.4)	9 (50)/9 (50)	0.11 ^§^
Height, m	1.22 ± 0.03	1.21 ± 0.04	0.53 ^‡^
Weight, kg	28.19 ± 2.56	28.42 ± 2.18	0.78 ^‡^
BMI, kg/m^2^	18.96 ± 1.33	19.43 ± 2.04	0.42 ^‡^
Pre-intervention measures of the dependent outcomes			
Dorsiflexor strength, newtons	16.02 ± 1.97	16.93 ± 2.45	0.23 ^‡^
Ankle’s AROM, degrees	12.36 ± 0.65	12.02 ± 0.79	0.16 ^‡^
10 mWT, s	1.45 ± 0.03	1.56 ± 0.05	0.10 ^‡^
6 MWT, m	378.80 ± 18.76	387.83 ± 16.35	0.13 ^‡^
TUG, s	14.54 ± 0.87	14.77 ± 1.24	0.52 ^‡^

CT: cross-training; b/g: boy/girl; RT: right; LT: left; MAS: Modified Ashworth Scale; 10 mWT: 10-meter walk test; 6 MWT: 6-min walk test; TUG: modified timed up-and-go; AROM: active range of motion. Categorical data are expressed as frequency (%), and numerical data are shown as mean ± standard deviation. *p* values: ^‡^ unpaired *t*-test, ^§^ Pearson’s χ^2^ test.

**Table 2:** Overview of inter- and intra-group variations in muscle strength and joint mobility metrics.

		Control Group (*n* = 18)	CT Group (*n* = 18)	Between-Subject Effect	
				*p*-Value	Partial η^2^
Dorsiflexor strength, newtons					
	Pre	16.02 ± 1.97	16.93 ± 2.45	0.032 *	0.128
	Post	17.93 ± 2.64	19.57 ± 1.66		
	*p*-value	0.03 *	<0.001 *		
	Hedges’ g (95% CI)	0.54 (0.04–1.02)	0.89 (0.34–1.44)		
Ankle’s AROM, degrees					
	Pre	12.36 ± 0.65	12.02 ± 0.79	0.016 *	0.159
	Post	13.16 ± 0.58	13.70 ± 0.67		
	*p*-value	<0.001 *	<0.001 *		
	Hedges’ g (95% CI)	1.30 (0.67–1.93)	2.89 (1.84–3.94)		

CT: cross-training; AROM: active range of motion; CI: confidence interval. Pre- and post-intervention data are shown as mean ± standard deviation. Partial *η*^2^: effect size of between-subject effects; Hedges’ *g*: *t*-test effect size. * Significant at *p* < 0.05.

**Table 3:** Comparative analysis of locomotor ability metrics (10 mWT, 6 MWT, and TUG) across and within the study groups.

		Control Group (*n* = 18)	CT Group (*n* = 18)	Between-Subject Effect	
				*p*-Value	Partial η^2^
10 mWT, m/s					
	Pre	1.45 ± 0.03	1.56 ± 0.05	0.017 *	0.157
	Post	1.48 ± 0.06	1.61 ± 0.06		
	*p*-value	<0.001 *	<0.001 *		
	Hedges’ g (95% CI)	2.01 (1.21–2.81)	2.68 (1.69–3.67)		
6 MWT, m					
	Pre	378.80 ± 18.76	387.83 ± 16.35	0.004 *	0.222
	Post	395.60 ± 12.39	408.00 ± 11.43		
	*p*-value	<0.001 *	<0.001 *		
	Hedges’ g (95% CI)	0.96 (0.40–1.52)	1.09 (0.51–1.68)		
TUG, s					
	Pre	14.54 ± 0.87	14.77 ± 1.24	0.047 *	0.111
	Post	13.60 ± 0.98	12.97 ± 0.85		
	*p*-value	0.01 *	<0.001 *		
	Hedges’ g (95% CI)	0.72 (0.20–1.23)	1.08 (0.50–1.67)		

CT: cross-training; 10 mWT: 10-m walk test; 6 MWT: 6-min walk test; TUG: modified timed up-and-go test; CI: confidence interval. Data are shown as mean ± standard deviation. Partial *η*^2^: effect size of between-subject effects; Hedges’ *g*: *t*-test effect size. * Significant at *p* ˂ 0.05.

## Data Availability

The data that support the findings of this study are available on reasonable request from the corresponding author.

## Abbreviations

The following abbreviations are used in this manuscript:

10 mWT	10-m walk test
6 MWT	6-min walk test
ANOVA	Univariate analysis of variance
AROM	Active range of motion
BMI	Body mass index
CP	Cerebral palsy
CT	Cross-training
HHD	Handheld dynamometer
MANOVA	Multivariate analysis of variance
SPSS	Statistical Package for the Social Sciences
TUG	Timed up-and-go test
UCP	Unilateral cerebral palsy
